# Integrating gross morphology and bone histology to assess skeletal maturity in early dinosauromorphs: new insights from *Dromomeron* (Archosauria: Dinosauromorpha)

**DOI:** 10.7717/peerj.6331

**Published:** 2019-02-11

**Authors:** Christopher T. Griffin, Lauren S. Bano, Alan H. Turner, Nathan D. Smith, Randall B. Irmis, Sterling J. Nesbitt

**Affiliations:** 1 Department of Geosciences, Virginia Tech, Blacksburg, VA, USA; 2 Department of Biology, Virginia Tech, Blacksburg, VA, USA; 3 Department of Anatomical Sciences, Stony Brook University, Stony Brook, NY, USA; 4 The Dinosaur Institute, Natural History Museum of Los Angeles County, Los Angeles, CA, USA; 5 Natural History Museum of Utah, University of Utah, Salt Lake City, UT, USA; 6 Department of Geology and Geophysics, University of Utah, Salt Lake City, UT, USA

**Keywords:** Ontogeny, Skeletal maturity, Histology, Dinosauromorph, Triassic, Bone scar

## Abstract

Understanding growth patterns is central to properly interpreting paleobiological signals in tetrapods, but assessing skeletal maturity in some extinct clades may be difficult when growth patterns are poorly constrained by a lack of ontogenetic series. To overcome this difficulty in assessing the maturity of extinct archosaurian reptiles—crocodylians, birds and their extinct relatives—many studies employ bone histology to observe indicators of the developmental stage reached by a given individual. However, the relationship between gross morphological and histological indicators of maturity has not been examined in most archosaurian groups. In this study, we examined the gross morphology of a hypothesized growth series of *Dromomeron romeri* femora (96.6–144.4 mm long), the first series of a non-dinosauriform dinosauromorph available for such a study. We also histologically sampled several individuals in this growth series. Previous studies reported that *D. romeri* lacks well-developed rugose muscle scars that appear during ontogeny in closely related dinosauromorph taxa, so integrating gross morphology and histological signal is needed to determine reliable maturity indicators for early bird-line archosaurs. We found that, although there are small, linear scars indicating muscle attachment sites across the femur, the only rugose muscle scar that appears during ontogeny is the attachment of the *M. caudofemoralis longus*, and only in the largest-sampled individual. This individual is also the only femur with histological indicators that asymptotic size had been reached, although smaller individuals possess some signal of decreasing growth rates (e.g., decreasing vascular density). The overall femoral bone histology of *D. romeri* is similar to that of other early bird-line archosaurs (e.g., woven-bone tissue, moderately to well-vascularized, longitudinal vascular canals). All these data indicate that the lack of well-developed femoral scars is autapomorphic for this species, not simply an indication of skeletal immaturity. We found no evidence of the high intraspecific variation present in early dinosaurs and other dinosauriforms, but a limited sample size of other early bird-line archosaur growth series make this tentative. The evolutionary history and phylogenetic signal of gross morphological features must be considered when assessing maturity in extinct archosaurs and their close relatives, and in some groups corroboration with bone histology or with better-known morphological characters is necessary.

## Introduction

Assessing the skeletal maturity of individuals of extinct taxa is critical to interpreting the paleobiology of their constituent species and clades. Interpretations of the growth stages of a taxon impact evaluations of species synonymy (Abdala & Giannini, 2000; Vucetich et al., 2005; Cartelle & De Iuliis, 2006; Billet, de Muizon & Mamani Quispe, 2008; Scannella & Horner, 2010; Shelton et al., 2012), potential sexual dimorphism (Raath, 1990; Klein & Sander, 2008; Saitta, 2015), maximum body size (e.g., Huttenlocker & Rega, 2012; Turner & Nesbitt, 2013), intraspecific variation (Brinkman, 1988; Raath, 1990; Griffin & Nesbitt, 2016a, 2016b), and ecological role (Codron et al., 2012), among others (Hone, Farke & Wedel, 2016). Reliable skeletal maturity indicators are present in many extant vertebrate groups, and these characteristics have been used successfully to estimate skeletal maturity and growth stage in their extinct members. For example, in placental mammals, the closure of epiphyseal sutures indicates cessation of growth (e.g., Dawson, 1925; Morris, 1972; Ogden, Conlogue & Rhodin, 1981; Storå, 2002; Nilsson & Baron, 2004; Köhler & Moyà-Solà, 2009; Moran et al., 2015) and the presence of permanent teeth can give an indication that the individual is not among the earliest stages of growth, when deciduous (or milk) teeth are present (e.g., Morris, 1972; Spinage, 1973; Smith, Crummett & Brandt, 1994). Among reptiles, many squamates also fuse epiphyses during ontogeny, which provides an indicator of body size at skeletal maturity in taxa with determinate growth (e.g., Haines, 1969; Chinsamy-Turan, 2005; Frýdlová et al., 2017). However, it remains challenging to assess the skeletal maturity of individuals from clades that are highly disparate or possess long evolutionary histories with highly modified living members. This difficulty is exemplified in archosaurs (crocodylians, birds, and their extinct relatives), which possess a larger range of body sizes than any other clade of terrestrial animals (Carrano, 2006; Benson et al., 2014; Turner & Nesbitt, 2013). In this clade, many indicators of skeletal maturity in other living reptiles are absent or of limited use (e.g., epiphyseal fusion, Chinsamy-Turan, 2005; Frýdlová et al., 2017), making determining skeletal maturity difficult (Hone, Farke & Wedel, 2016).

To overcome this dilemma, many studies have employed a variety of assessment techniques derived from a combination of observations in living taxa and deductions made from extinct taxa. The use of bone histology in extinct archosaurs (e.g., Horner, de Ricqlès & Padian, 2000; Padian, Horner & de Ricqlès, 2004; Erickson, 2014) aids in determining an individual’s skeletal maturity through the presence or absence of lines of arrested growth (LAGs) as well as the pattern of tissue organization (e.g., collagen fiber/hydroxyapatite crystal density, fiber orientation) and vascularity present in the bone (reviewed in Huttenlocker, Woodward & Hall, 2013). In addition to its success in providing indicators that asymptotic size has been reached (e.g., Woodward, Horner & Farlow, 2011; Erickson et al., 2004; 2007; Erickson, 2014), studies of bone histology have contributed to answering a diverse array of questions, including determining the thermometabolism of extinct archosaurs (e.g., Legendre et al., 2016), hypothesizing sex-specific tissues (e.g., Schweitzer, Wittmeyer & Horner, 2005; 2016; Lee & Werning, 2008) and estimating age of sexual maturity in dinosaurs (Lee & Werning, 2008), estimation of growth rates (e.g., Cubo et al., 2012), and hypothesizing behavioral implications of embryonic and prenatal development (Horner, Padian & de Ricqlès, 2001). However, creating osteohistological thin sections is time consuming and permanently alters original bone morphology through consumptive sampling, and so this method may not be viable for rare species and for specimens with exceptional preservation. Furthermore, some specimens comprise a taphonomic mode that may not preserve internal structure (e.g., Jans, 2008; Pewkliang, Pring & Brugger, 2008). Depending on the taxon, osteohistological thin sections may also lack cyclical indicators of numerical ontogenetic age (e.g., LAG), making these data ambiguous or of limited utility when used independently of other methods (e.g., the silesaurid *Asilisaurus kongwe*, Griffin & Nesbitt, 2016a; several sauropod dinosaurs, Sander et al., 2004). Therefore, although osteohistology is a powerful tool for understanding growth both in terms of absolute and relative ontogenetic age, its potential drawbacks necessitate other means of constraining maturity assessment.

In contrast with osteohistological age assessment, gross morphological indicators of skeletal maturity provide an immediate and nondestructive assessment of the ontogenetic stage of an individual. Skeletal proportions (Bennett, 2001; Piechowski, Tałanda & Dzik, 2014), periosteal surface texture (Sampson, Ryan & Tanke, 1997; Tumarkin-Deratzian, Vann & Dodson, 2006, 2007; Brown, Russell & Ryan, 2009), suture closure (Brochu, 1996; Irmis, 2007; Bailleul et al., 2016), muscle scarring (Raath, 1990; Tumarkin-Deratzian, Vann & Dodson, 2006, 2007; Griffin & Nesbitt, 2016a, 2016b), and clade specific indicators (e.g., growth of ceratopsian ornamentation; Hone, Farke & Wedel, 2016) have all been used to assess skeletal maturity in extinct archosaurs, especially non-avian dinosaurs. However, these methods are not always applicable across clades, which can produce results difficult to assess independently. For example, variable timing of the closure of sutures, a commonly used method for assessing skeletal maturity in archosaurs (e.g., vertebral neurocentral sutures, Brochu, 1996; Irmis, 2007) can be influenced by changes in biomechanics (e.g., in cranial sutures, Bailleul et al., 2016), and the sequence of postcranial co-ossification events can be variable both between clades (Irmis, 2007) and within a single species (Griffin, 2018). Therefore, skeletal maturity assessment using morphological characteristics alone may result in ambiguous age determinations. In addition, taken alone, these methods are nearly always relative age indicators; without independent evidence from other sources (e.g., histology), they can only indicate whether one individual is older or younger than another.

The strength of a well-supported assessment of skeletal maturity lies in the combination of external morphological and histological data. Yet the relationship between histological and morphological maturity has not been critically examined in most archosaurian clades (but see Woodruff, Fowler & Horner, 2017 for this combination of methods in diplodocids). This integration is especially important in examining growth of early dinosaurs and their close relatives, which possess high intraspecific variation in growth (Griffin & Nesbitt, 2016a, 2016b). This high variation has been almost entirely observed through the lens of gross morphology (Griffin & Nesbitt, 2016a, 2016b; Barta, Nesbitt & Norell, 2017; Griffin, 2018), whereas histology has only been touched on in a small number of taxa (Griffin & Nesbitt, 2016a). Therefore, the origin of this variation and the relationship between gross morphological and histological ontogenetic changes is poorly constrained by a lack of integration between methods of maturity assessment in early bird-line archosaurs.

In this study, we examined the histology and gross morphology of the first available femoral growth series of a non-dinosauriform dinosauromorph to better understand the origin of the dinosaurian growth pattern. We assess the skeletal maturity of well-preserved femora of *Dromomeron romeri*, an early-diverging dinosauromorph from the Late Triassic Period of northern New Mexico, USA (Irmis et al., 2007; Nesbitt et al., 2009a), using well-developed bone scars, which typically indicate muscle, ligament, and tendon attachment sites. These femoral features are important morphological characteristics that have been used for determining skeletal maturity in previous studies of early dinosauromorphs (Raath, 1990; Nesbitt et al., 2009a; Piechowski, Tałanda & Dzik, 2014; Griffin & Nesbitt, 2016a, 2016b). Although these scars appear sequentially throughout ontogeny (Nesbitt et al., 2009a; Griffin & Nesbitt, 2016a, 2016b), how the development of bone scars relates to absolute ontogenetic age—and how widespread the utility of these scars are for assessing skeletal maturity among early bird-line archosaurs—is poorly constrained by a lack of ontogenetic series. Previous studies indicate that *D. romeri* lacks well-developed bone scars that other closely related archosaurs develop in the proximal region of the femur (Irmis et al., 2007; Nesbitt et al., 2009a; Martínez et al., 2016; Cabreira et al., 2016). In this study, we determine whether this lack of femoral scarring is indicative of the skeletal immaturity of the specimens (Bennett, 2015), or if this absence is an evolutionary novelty (i.e., autapomorphy). In doing so we integrated histological and gross morphological signals to better understand their relationship between bone scarring, ontogenetic age, and evolutionary history in early archosaurs.

## Materials and methods

### Taxonomic justification

We consider all femoral specimens examined in this study to be assignable to *D. romeri* based on the shared presence of diagnostic character states of the referred material and the holotype femur (e.g., absence of a distinct ridge for the attachment of the *M. caudofemoralis longus* (this absence is not ontogenetic, see below); presence of a ridge on the anteromedial edge of the distal end; Irmis et al., 2007; Nesbitt et al., 2009a) and these specimens’ provenance (all were collected from the type locality—Hayden Quarry, Ghost Ranch, NM, USA). The tibia and fibula of GR 238 are identifiable as *D. romeri* because they were found articulated with each other and with the femur of an individual diagnosable as *D. romeri*, even though the proximal and distal ends of these elements are not preserved.

Bennett (2015) recently suggested that the holotype of *D. romeri*, an isolated femur (GR 218), is a skeletally immature individual of the theropod *Tawa hallae* based on femoral size, the absence of a fourth trochanter interpreted as indicative of skeletal immaturity, and the suggestion that the morphology of the proximal and distal ends of the femur are incompletely ossified. However, the smallest *Tawa hallae* femur (116.3 mm in length; GR 244) is smaller than the larger *D. romeri* femora in our study (e.g., 144.4 mm in length, GR 1038; 135.3 mm in length, GR 238), and all recent cladistic analyses including early dinosauromorphs have recovered *D. romeri* as a lagerpetid dinosauromorph (Irmis et al., 2007; Nesbitt et al., 2009a; 2009b; Nesbitt, 2011; Martínez et al., 2016; Cabreira et al., 2016; Müller, Langer & Dias-da-Silva, 2018). Additionally, the postcranium of an associated specimen of *D. romeri* (GR 1041) lacks dinosaurian and dinosauriform synapomorphies, instead possessing apomorphic character states that pertain to lagerpetids—outside Dinosauriformes—and these character optimizations have been borne out in all phylogenetic analyses. Specifically, *D. romeri* lacks an ilium with a convex ventral acetabular margin (= “imperforate” acetabulum, character 273–0, Nesbitt, 2011; GR 1041), a fibular condyle of the tibia that is level with the medial condyle at its caudal border (character 201–1 from Cabreira et al., 2016; character from Langer & Benton, 2006), and a posterior groove present on the astragalus (character 217–1 from Cabreira et al., 2016; character from Nesbitt, 2011). In addition, *D. romeri* has been independently supported as a unique taxon of non-dinosaurian dinosauromorph based on the large number of differences in femoral characters between *D. romeri* and *Tawa hallae* compared to ontogenetic series of other early dinosauromorphs (Müller, 2017). Thus, all current evidence strongly supports the original division of the two taxa and we concur with Müller (2017) in considering *D. romeri* to be a non-dinosaurian dinosauromorph. Nonetheless, our examination here of the gross morphological and histological ontogeny of *D. romeri* provides further evidence to test Bennett’s (2015) hypothesis. We can falsify this hypothesis if any *Dromomeron* specimens are demonstrated to be near skeletal maturity.

### Gross morphological assessment and histological sampling

Seven femora were included in our *Dromomeron romeri* sample, hypothesized to form an ontogenetic series (GR 218, 234, 238, 1036–1039; femur length between 96.6 and 144.4 mm; Table 1). We use the term ‘well-developed bone scar’ to refer to rugose scars of muscle, tendon, or ligament attachment that are distinct from the shaft or main body of the bone. The term ‘poorly developed bone scar’ refers to scars of attachment which do not comprise rugose processes or features distinct form the shaft, such as thin lineations or grooves left by muscle attachments on bone. Poorly developed bone scars can be useful for hypothesizing muscle attachment sites but are not as informative for assessing maturity (e.g., Griffin & Nesbitt, 2016a). We evaluated the femora for presence of muscle scars by comparing the position of femoral muscle origins and insertions in extant Aves and Crocodylia, those inferred in dinosaurs and relatives (Rowe, 1986; Hutchinson, 2001), and in closely related taxa [i.e., studies on *Dromomeron gregorii* (Nesbitt et al., 2009a); *D. gigas* (Martínez et al., 2016); *Ixalerpeton polesinensis* ( Cabreira et al., 2016); *Asilisaurus kongwe* (Griffin & Nesbitt, 2016a); *Teleocrater rhadinus* (Nesbitt et al., 2017)]. We whitened specimens for high-contrast photography of bone scars using removable ammonium chloride following standard techniques (Feldmann, 1989; Green, 2001; Hegna, 2010; Shelburne & Thompson, 2016). After coating the specimen in a thin layer of B-72 glue dissolved in acetone, we heated the ammonium chloride in a glass test tube and held the specimen under the resulting vapor stream until the area of interest was satisfactorily whitened. Each specimen was photographed with a Canon EOS Rebel t4i digital camera. Following photography, the ammonium chloride was removed with a gentle stream of water. We used a macro lens (Tamron AF 90mm f/2.8 Di SP AF/MF 1:1 Macro Lens) for close-up photography of these bone scars, and photographs taken in stacks of different focal planes were blended in Adobe Photoshop CS6 v. 13.0.6.

**Table 1:** Specimens of *Dromomeron romeri* used in this study with measurements in mm.

Specimen	Femoral length	Maximum proximal width	Maximum distal width
GR 1037	96.6	20.1	18.4
GR 218	96.9	18.7	18.3
GR 1036*	103.9	18.9	18.5
GR 234	126.8	23.1	23.3
GR 238*	135.3	23.3	26.7
GR 1039	135.5	30	26.7
GR 1038*	144.4	28.5	26.3

**Note:**

Asterisks (*) indicate specimens that were histologically sampled.

One isolated femur (GR 1036), a tibia and fibula from the largest articulated individual (GR 238), and the largest femur (GR 1038) were histologically sampled as close to the midshaft region as possible. The midshaft region of the bone is generally consistent in the type of tissue present and does not remodel as much as other parts of the bone; the periosteal surface is also older, and therefore has more recorded information when compared to the distal and proximal ends (Padian, Lamm & Werning, 2013). To preserve the original gross morphology of the specimens before destructive sampling, we molded (using Smooth-On Mold Star 15 SLOW platinum silicone rubber) and casted (using Smooth-On Smooth Cast 300 liquid plastic resin) each specimen before thin sectioning. The tibia and fibula of GR 238 were sampled because the femora of this individual were not available for histological sectioning. Because the largest femur (GR 1038) was from the largest known *D. romeri* individual, and was well-preserved and complete, we were unable to section this element fully. To collect data on the histological maturity of this individual, we instead removed a portion of the midshaft which included the outermost cortex, rather than cut the entire element in half. Although we were not sure if this sample contained a complete record of the cortex when we sampled this element, we have evidence for the presence of endosteal lamellae in the internalmost portion of the sample (see Results), indicating that at least a portion of this sampled bone fragment spans the entire cortex. We created two histological slides from this portion, with slide 1 being the more proximal section of the two.

We embedded the sampled bone in Castolite AC clear polyester resin (Eager Polymers) and placed it in a vacuum chamber for 2–3 min to eliminate bubbles before curing overnight. Thin wafers (1.5 mm) were cut from the original sample perpendicular to the proximodistal axis with an IsoMet 1000 precision saw. We ground down one side, starting with a 1200 grit grinding disc and ending with a 2400 grit disk; the wafer was then polished with 0.3 micron slurry powder. We then glued the polished side face down on a slide and the opposite side was ground and polished to the desired thickness. Finally, we examined the slides with an Olympus BX51 petrographic microscope with plane- and cross-polarized light, using a gypsum wedge (530 nm) to better observe the orientations of hydroxyapatite crystallites (which are in turn influenced by the orientation of the original collagen fibrils). Images were captured with an Infinity1 camera and associated software. We also captured high-resolution images of the full slides using a Nikon Eclipse LV100ND transmitted and reflected light microscope, imaging them using a Nikon DS-Fi2 camera and Digital sight DS-U3 interface together with a Prior ProScan III automated microscope stage and digitally assembled using Nikon NIS-Element Basic Research v. 4.40.00 (Build 1084). These full-slide images were captured in three light regimes: plane-polarized light, cross-polarized light, and cross-polarized light with a gypsum plate (530 nm). The exception to this was the histology of the largest femur, GR 1038, which was examined with an Olympus BX51 research microscope under plane- and cross-polarized light (with and without a gypsum plate), and images were captured with Lumenera Infinity capture imaging software. Large high-resolution whole-slide images have been uploaded onto the online repository Morphobank (Project Number 3127: http://morphobank.org/permalink/?P3127).

## Results

### Gross morphology

**Figure 1:**
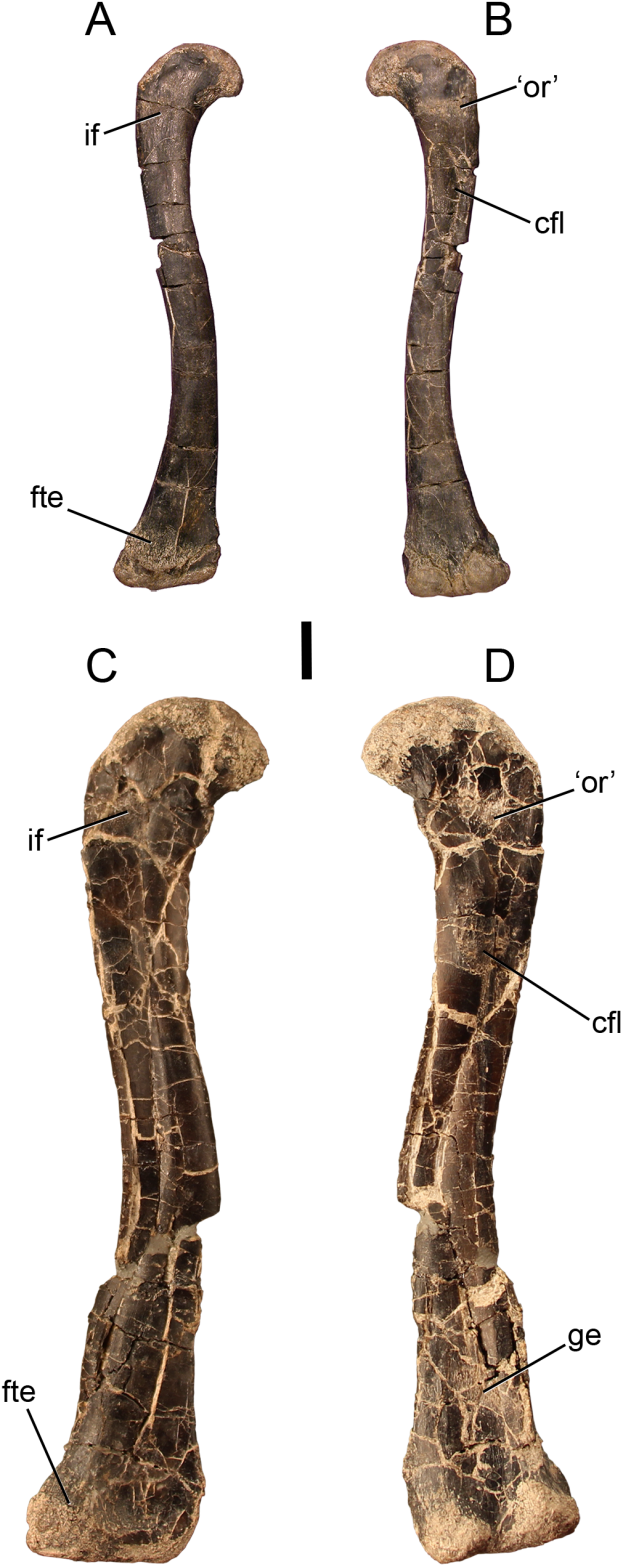
The smallest and largest femora of *Dromomeron romeri*, which are also the best preserved. The left femur GR 218 (the holotype) in (A) anterolateral and (B) posteromedial views. Note that this specimen has been mirrored for easy comparison. The right femur GR 1308 in (C) anterolateral and (D) posteromedial views. Scale bar is 1 cm. Abbreviations: cfl, attachment of the *M. caudofemoralis longus*; fte, attachment of the *M. femorotibialis externus*; ge, attachment of the *M. gastrocnemius externus*; if, attachment of the *M. iliofemoralis*; ‘or’, the homolog of the dinosaurian ‘obturator ridge’. Photographs by Sterling Nesbitt and Christopher Griffin.

**Figure 2:**
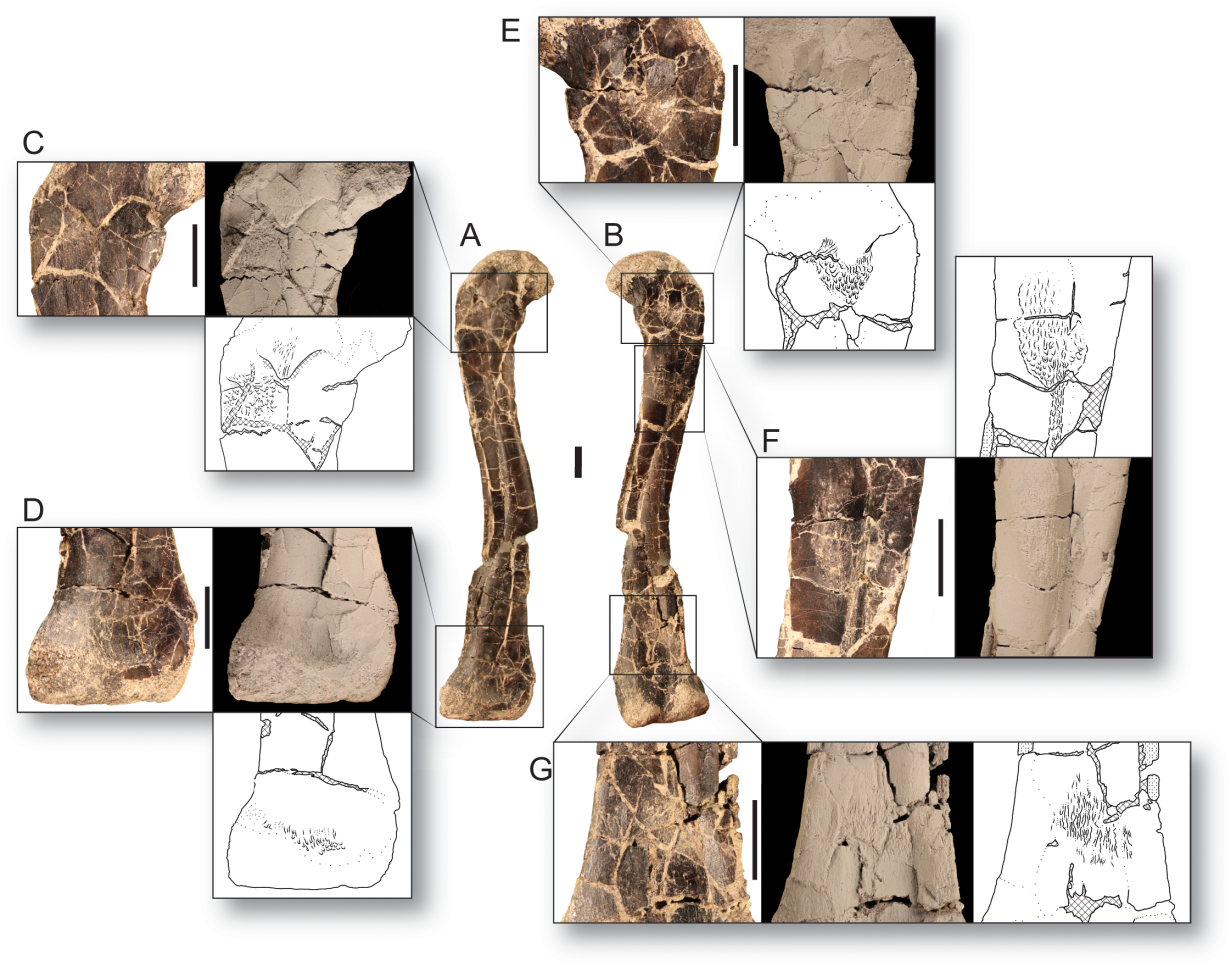
Bone scars of the largest femur of *Dromomeron romeri* in (A) anterolateral and (B) posteromedial views. (C) The attachment of the *M. iliofemoralis* in standard view, whitened, and in line drawing. (D) The attachment of the *M. femorotibialis externus* in standard view, whitened, and in line drawing. (E) The ‘obturator ridge’ in standard view, whitened, and in line drawing. (F) The well-developed attachment scar of the *M. caudofemoralis longus* in standard view, whitened, and in line drawing. (G) The attachment of the *M. gastrocnemius externus* in standard view, whitened, and in line drawing. Thin lineations in all line drawings indicate muscle scars. GR 1308 is a right femur. Scale bars are 1 cm. All photographs and line drawings by Christopher Griffin.

Well-developed scars just distal to the proximal articular surface of the femur do not appear in our sample of *Dromomeron romeri* individuals (Figs. 1 and 2). Instead, thin lineations of striated bone (i.e., poorly developed scars) mark what we hypothesize to be the insertion of the *M. iliotrochantericus caudalis* on the anterolateral surface of the proximal end of the femur. This muscle has been interpreted to form the anterior trochanter in many other early bird-line archosaurs (Hutchinson, 2001; Maidment & Barrett, 2011), which appears during ontogeny in several early dinosauromorphs (*D. gregorii*, Nesbitt et al., 2009a; *Asilisaurus kongwe*, Griffin & Nesbitt, 2016a). This poorly developed scar is in a similar location as the interpreted insertion of the *M. iliofemoralis externus* in the non-ornithodiran avemetatarsalian *Teleocrater rhadinus* (Nesbitt et al., 2017; note that the insertions of the *M. iliofemoralis externus* [= trochanteric shelf] and the *M. iliotrochantericus caudalis* [= anterior trochanter] are mistakenly reversed in Nesbitt et al., 2017, but corrected in Nesbitt et al., 2018). Larger femora of *D. gregorii* also possess a continuous lightly striated structure which is hypothesized to correspond to both the anterior trochanter and trochanteric shelf (Nesbitt et al., 2009a).

Unlike the other scars on the proximal end of the femur, the attachment site morphology of the *M. caudofemoralis longus* insertion (= fourth trochanter in most archosaurs, Hutchinson, 2001; Maidment & Barrett, 2011; Schachner, Manning & Dodson, 2011) does vary in our sample of *D. romeri* (Figs. 1 and 2C). All individuals in this series lack the ridge-like fourth trochanter found in many other lagerpetid taxa (*D. gregorii*, Nesbitt et al., 2009a; *Lagerpeton chanarensis*, Sereno & Arcucci, 1994; *Ixalerpeton polesinensis*, Cabreira et al., 2016). However, the largest femur in our sample (GR 1038) possesses a rugose scar marking the insertion of the *M. caudofemoralis longus* (Fig. 2D), with similar morphology to the homologous scar in *D. gigas* (Martínez et al., 2016). However, unlike *D. gigas*, *D. romeri* shows no evidence of a posteromedial scar (sensu Martínez et al., 2016), which may be homologous with the ‘obturator ridge’ of early dinosauriforms and dinosaurs (Griffin, 2018). Nonetheless, *D. romeri* does possess poorly developed scars on this portion of the femur, which probably mark the attachment of the muscle(s) or ligament(s) that form the ‘obturator ridge’ of *D. gigas* and dinosauriforms. Therefore, only a single well-developed muscle scar, the insertion of the *M. caudofemoralis longus*, appears during ontogeny in *D. romeri*; it does not form a flange or ridge-like fourth trochanter as present in almost all other early diverging dinosauromorphs and early archosaurs (cf. Nesbitt, 2011: Figs. 37–39).

The distal end of the femur also possesses lineations that indicate muscle attachment sites (Figs. 1 and 2). The anterior of the distalmost portion of the femur, just proximal to the unfinished bone of the distal articular surface, possesses a rough, linearly grooved area that marks the attachment of *M. femorotibialis externus* (following the hypothesis of Nesbitt et al., 2009a). This scar is present in all femora of *D. romeri* for which its presence can be evaluated, including the smallest specimen (GR 218; Fig. 1), and is also present in larger individuals of *D. gregorii* (Nesbitt et al., 2009a) as well as in *D. gigas* (Martínez et al., 2016); this scar has been hypothesized to be a synapomorphy of the clade *Dromomeron* (Nesbitt et al., 2009a; Müller, Langer & Dias-da-Silva, 2018: character state 194-1).

Another poorly developed scar extends in an anteroproximal to posterodistal orientation across the posterior side of the femur, just proximal to the popliteal surface (Fig. 1D). This scar is subtle and unable to be evaluated in all specimens of *D. romeri* because of preservation quality; however, two of the smaller individuals lack this scar (GR 218, 1037; Fig. 1B), and it is present in the two largest individuals for which it can be evaluated (GR 234, 1038; Figs. 1D and 2E). This suggests that this scar may appear during ontogeny, although the condition in all *D. romeri* specimens greatly differs from the well-developed scars typical of archosaurian ontogeny (e.g., anterior trochanter, fourth trochanter, trochanteric shelf; Raath, 1990; Tumarkin-Deratzian, Vann & Dodson, 2006, 2007; Griffin & Nesbitt, 2016a, 2016b). This small posterior femoral scar appears to also be present in the femora of other lagerpetids, including *D. gregorii* (Nesbitt et al., 2009a: Fig. 2B) and *D. gigas* (Martínez et al., 2016: Fig. 2.2), and potentially *Lagerpeton chanarensis* (Nesbitt et al., 2009a: Fig. 3B). Based on its location in comparison to *Alligator* and Aves (Carrano & Hutchinson, 2002), we hypothesize that this scar is an attachment for *M. gastrocnemius externus* (= *Mm. gastrocnemii pars lateralis* of Aves; Carrano & Hutchinson, 2002).

### Histology

For osteohistological descriptions, we use the terminology of Werning (2013), who expanded on and provided diagnostic criteria for the terminology of Francillon-Vieillot et al. (1990). Because of uncertainty in identification given the intermediate level of tissue organization present in many of these sections (see below) and the fact that we have no longitudinally sampled slides to diagnose woven-fibered bone with the highest certainty (Stein & Prondvai, 2014; Prondvai et al., 2014), we do not refer to woven- or parallel-fibered tissue with lamellated primary osteons as a ‘fibrolamellar complex’, but instead describe the tissue organization and vascular orientation/style separately.

**Figure 3:**
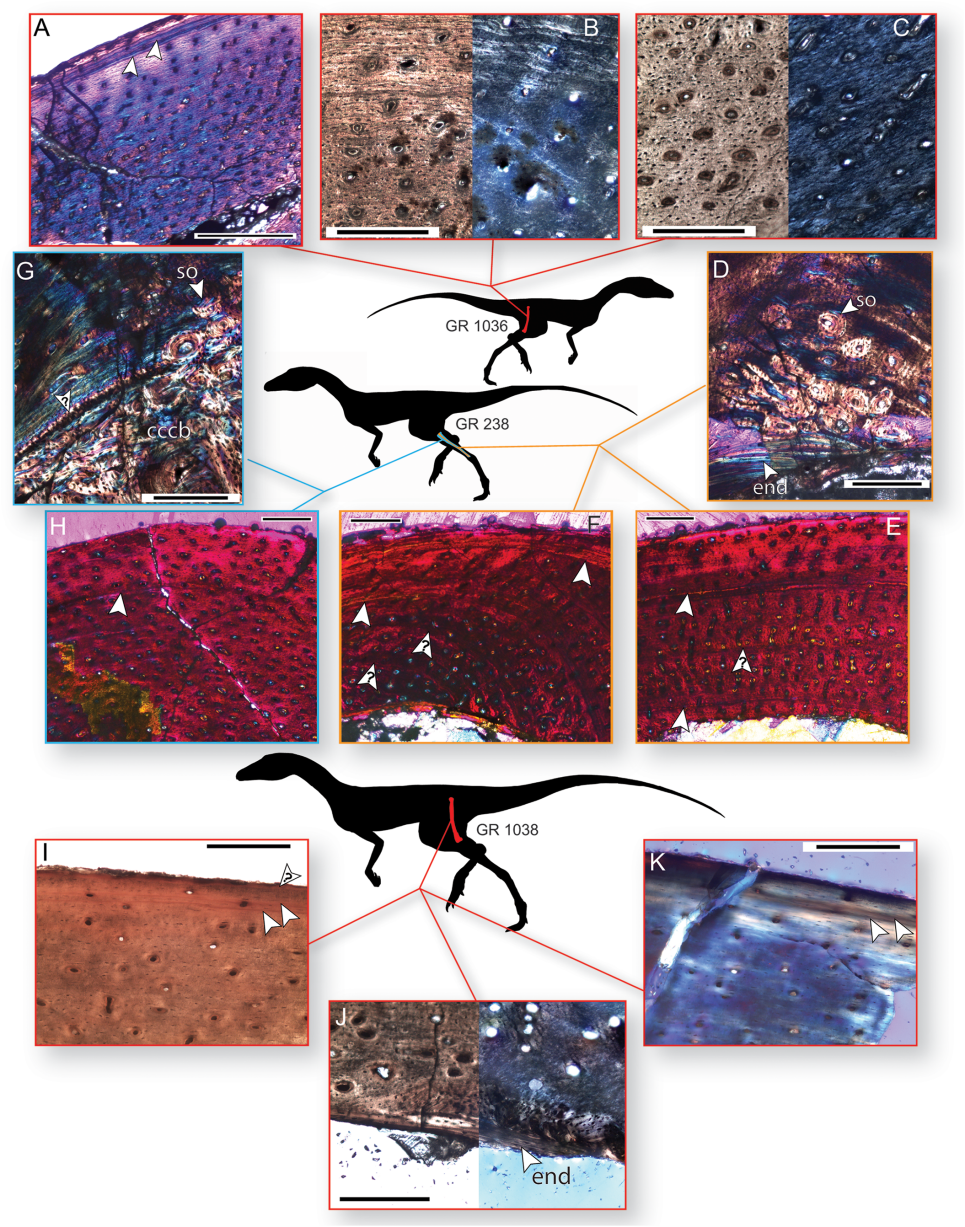
Bone histology of three individuals of *Dromomeron romeri*. Silhouettes are modified from Cabreira et al. (2016: Fig. 1A). (A) Femoral histology of the smaller sampled individual (GR 1036) under cross-polarized light with a gypsum waveplate, showing two LAGs near the periosteal surface. (B) Femoral histology of GR 1036 under plane-polarized and cross-polarized light showing the transition from less- to more-organized tissue in the external third of the cortex. (C) Femoral histology of GR 1036 showing tissues typical of the less-organized primary bone of the internal two-thirds of the cortex, under plane-polarized and cross-polarized light. (D) Fibular histology of the mid-sized sampled individual (GR 238) under cross-polarized light with a gypsum waveplate, showing the boundary of the medullary bone with endosteal lamellae, secondary osteons, and highly organized primary bone. (E) Fibular histology of GR 238 under cross-polarized light with a gypsum waveplate showing two LAGs and the periosteal surface. (F) Fibular histology of GR 238 under cross-polarized light with a gypsum waveplate showing two LAGs and the periosteal surface. (G) Tibial histology of the mid-sized sampled individual (GR 238) under cross-polarized light with a gypsum waveplate showing internal compacted coarse cancellous bone, external primary parallel-fibered bone, and the boundary between these two tissues (an osteocyte-rich anulus or growth line, marked with a ‘?’. Note secondary osteons in the internalmost portion of the parallel-fibered bone. (H) Tibial histology of the mid-sized sampled individual (GR 238) under cross-polarized light with a gypsum waveplate. (I) The subperiosteal surface of the femoral histology of the largest individual (GR 1038) under plane-polarized light. (J) The internal portion of the sample cortex of the largest individual (GR 1038) under plane-polarized and cross-polarized light, showing endosteal lamellae and internal, osteocyte-rich woven bone that grades into parallel-fibered bone externally. Abbreviations: cccb, compacted coarse cancellous bone; end, endosteal lamellae; so, secondary osteon(s) Arrow indicates a partially vascularized annulus. (K) The subperiosteal surface of the femoral histology of the largest individual (GR 1038) under cross-polarized light with a gypsum waveplate. All unlabeled arrows indicate lines of arrested growth (LAGs); arrows with question marks indicate growth lines of uncertain identify­. The periosteal surface is always oriented up. Scale bars are 200 μm for A–D, G, I–K, and 250 μm for E–H. All photographs by Lauren Bano and Christopher Griffin.

The smaller of the two histologically sampled femora (GR 1036) is partially crushed, but sections of cortical bone preserving the entire endosteal-periosteal cross section are present. This bone comprises woven-fibered bone with primary osteons in the interiormost one-half to two-thirds of the cortex (in what is often termed a ‘fibrolamellar complex’, but see Stein & Prondvai, 2014). The apatite crystals in this region are more organized than stereotypical woven-fibered bone (Figs. 3A–3C), and may be considered intermediate between ‘classic’ woven-fibered bone and highly organized parallel-fibered bone (sensu Stein & Prondvai, 2014). Consistent with this level of organization, the osteocyte lacunae in this region are preferentially oriented circumferentially in the interior portion of the cortex, although many lacunae possess different orientations throughout this region. The internal, woven-fibered portion of the cortex is well-vascularized with primary osteons possessing one to two thin bands of concentric lamellae. These vascular canals are small (~10–30 µm in diameter) and circular, although some are slightly circumferentially oblong, and almost all are longitudinally oriented in the interiormost portion of the bone. Some radially oriented canals are present in the more external portions of the woven-fibered bone, and these canals are oriented at a slight angle (~25–30°) to the classic radial orientation, in which canals extend directly towards the subperiosteal surface. All these radial canals are angled in the same direction and to the same degree. Endosteal lamellae are present where the endosteal border is preserved. Roughly one-half to two-thirds of the distance to the subperiosteal surface, the woven-fibered bone smoothly transitions to highly organized parallel-fibered bone, and after this transition vascular density noticeably decreases to moderately well vascularized, or even poorly vascularized just internal to the subperiosteal surface (Figs. 3A and 3B). These vascular canals are primary osteons, and most are longitudinally oriented in this region with only one layer of lamellar bone ringing the canal. All osteocyte lacunae are elongate and oriented circumferentially in bands following the orientation of the collagen fibers. A LAG is present ~100 µm beneath the subperiosteal surface, and in portions of the bone this presents as an annulus or a double LAG (Fig. 3A). However, in this double LAG the interior LAG is more distinct than the outer LAG. There is no external fundamental system (EFS, a group of tightly spaced LAGs at the subperiosteal surface indicative that asymptotic size has been reached), and poorly vascularized parallel-fibered bone is present between the LAG and the subperiosteal surface (Fig. 3A).

The cortical bone of the tibia of GR 238 is broadly similar to that of the external tissues of the smaller femoral specimen (GR 1036) in that it comprises parallel-fibered primary bone with longitudinally oriented osteons (Figs. 3G and 3H). However, unlike the smaller femur (GR 1036), there are no radial canals. The vascular canals are similarly sized (~10–30 µm in diameter) throughout and possess one to two lamellae. Vascular density decreases in the external portion of the cortex, shifting from well-vascularized to between well- and moderately-well vascularized about three-fourths of the distance to the subperiosteal surface. A portion of the cortex on the medial side of the tibia differs from the rest of the sample bone; here, the parallel-fibered bone is less organized and less well-vascularized, the vascular canals are both circular and elliptical in shape, and the vascular density (i.e., number of vascular canals in a given area) decreases whereas the size of the vascular canals increases (40–100 µm in long-axis diameter; see online supplementary full-slide images on Morphobank). Osteocyte lacunae are denser than in the small femur, and only a small majority of lacunae are preferentially oriented circumferentially (and these preferentially oriented lacunae are in the internalmost cortex; Fig. 3G). The size and preferred orientation of osteocyte lacunae does not change between internal and external cortical bone. There are between one and two LAGs present in the sections of this element. The interior LAG is the most clearly defined, and is only ~150 µm away from the medullary border because of cortical drift; it has been eliminated by cortical drift in many portions of the cortex. The external LAG is less clearly defined (Fig. 3H) and is not easily traceable to different regions of the cortex. There is highly disorganized woven-fibered bone with large (100–150 µm in diameter) primary osteons internal to the deepest primary cortical bone on the medial and lateral sides of the element (Fig. 3G). Although the large osteons may superficially resemble secondary or Haversian bone, this is compacted coarse cancellous bone (CCCB; e.g., Chinsamy, 1995; Reid, 1996; Chinsamy-Turan, 2005) and is the result of cortical drift, as evidenced by a lack of cement lines around osteons (compare with cortical drift in the tibial histology of the silesaurid *A. kongwe*, Griffin & Nesbitt, 2016a: Fig. 4G). A clear boundary separates the CCCB from the more external primary cortical bone (Fig. 3G); however, in most regions this boundary is a simple difference in tissue types, with only one portion of the boundary is formed by a band of avascular, lacunae-rich bone (similar to an annulus). Osteocyte lacunae in the CCCB are large relative to the rest of the cortex and have no preferred orientation. Some trabecular fragments are preserved in the medullary cavity.

The sampled portion of the right fibula of GR 238 is comprised of predominantly primary parallel-fibered bone with primary osteons (generally with one lamellae), of overall similar composition, vascular density, and apatite crystallite organization to the tibia of this individual (Figs. 3D–3F). Unlike in the histology of the tibia, anastomosing vascular canals are not uncommon, although longitudinal canals still predominate, and these anastomoses impart a radial presentation to the canals (Figs. 3E and 3F). There are at least two LAGs that are traceable around the cortex except for a portion of the slide that has been ground too thin, and a portion where the internalmost LAG has been destroyed by medullary cavity migration/expansion. External to the second LAG, vascular density and connectivity decreases, and collagen fibers become more organized, with osteocytes oriented circumferentially along lamellae, although the cortex remains vascularized all the way to the edge of the subperiosteal surface (Figs. 3E and 3F). In the posterior and posteromedial portions of the cortex this decrease in vascular density and increase in organization is dramatic, with almost no vascular canals present, two additional LAGs visible, and highly organized parallel-fibered or even lamellar bone tissue containing abundant Sharpey’s fibers (Fig. 3D; Sharpey’s fibers not visible in figure, which focuses on the internal cortex; see online supplementary full-slide images on Morphobank). In addition to the LAGs, there are several weakly developed growth lines in the fibular cortex that do not persist around the entire circumference of the cortex (Figs. 3E and 3F); these may be either weak annual cycles or growth subcycles within a year constrained by true LAGs. The endosteal margin in this region also preserves several bands of avascular bone separating the medullary cavity from the cortical bone (Fig. 3D), although in other regions medullary cavity expansion or migration has apparently destroyed this avascular bone (Fig. 3E). A few secondary osteons are present just external to the endosteal margin on the posteromedial portion of the cortex (Fig. 3D).

The overall histology of the largest femur (GR 1038) is similar to that of the other sampled elements (Figs. 3l–3K). The majority of the sampled cortex is comprised of parallel-fibered primary bone, but the tissues possess an overall greater degree of organization than do those of the other elements. For example, most osteocyte lacunae are preferentially oriented in a circumferential direction except those of the internalmost sampled cortex (Figs. 3I–3K). Almost all vascular canals are ringed by a single layer of lamellar bone, and the vascular canals are almost exclusively longitudinal. Although the piece of cortex sampled from GR 1038 was an overhanging portion of the crushed midshaft, we hypothesize that the entire cortex, from endosteal lamellae to periosteal surface, is present in this sample. The internalmost portion of the sample in both slides 1 and 2 of GR 1038 comprises dense lamellar bone with large, globular osteocyte lacunae (Fig. 3J). This lamellar bone contains some simple vascular canals, and the interiormost surface is roughly parallel to the outermost surface where this lamellar bone is preserved. In slide 2, there is a small patch of more disorganized woven-fibered bone that abuts this lamellar bone on the internal side of the patch and grades into the normal cortical bone on the other (Fig. 3J). This lamellar bone possesses morphology consistent with that of endosteal lamellae. The vascular canals contained within the lamellar bone are also consistent with endosteal lamellae, as with several examples of vascularized endosteal lamellae that have recently been reported (Chinsamy, Cerda & Powell, 2016; Canoville et al., 2017; Prondvai et al., 2018). The line between the lamellar bone and the rest of the cortex is scalloped, indicating it was a resorption line on which the endosteal lamellae were then deposited, not smooth as would be expected in a LAG. In the outermost cortex the bone shifts to a highly organized parallel-fibered bone with decreased vascularization and circumferentially oriented apatite crystallites (Figs. 3I and 3K). Osteocyte lacunae are exclusively circumferentially oriented and elongate. There are two closely spaced LAGs within this parallel-fibered bone in the outermost region of the cortex, similar to GR 1036, and there appears to be a third LAG just beneath the subperiosteal surface (Fig. 3I). There are no secondary osteons in the sampled region of GR 1038, and no LAGs observed in the main body of the cortex.

## Discussion

### Growth of *Dromomeron romeri*

There are no large, robust muscle scars present across the sampled size range of *Dromomeron romeri* femora, although there are clear lineations showing the locations of muscle attachments. The sole exception to this is the attachment of the *M. caudofemoralis* musculature, which in most other early archosaurs forms a large flange- or wing-like structure (= fourth trochanter; Hutchinson, 2001; Nesbitt, 2011) but in *D. romeri* only forms a small scar, and only in the largest individual. Therefore, we hypothesize that *D. romeri* did form a well-developed femoral scar through ontogeny, although only one muscle attachment—that of the *M. caudofemoralis*—changed during development. There is no evidence for the development of other well-developed femoral scars in *D. romeri*; however, without considering histological data this absence could merely indicate skeletal immaturity in the individuals sampled.

The histology of the three individuals sampled suggest that *D. romeri* reached the larger sizes in our sample within at least one to 2 years of growth, although earlier LAGs may easily have been destroyed by medullary cavity expansion, or not recorded (i.e., no slow-down in growth), making this estimate a lower bound on the absolute ontogenetic age of these individuals. Neither of the smaller individuals had reached asymptotic size and were still growing, as indicated by a lack of an EFS in all elements sampled (Cormack, 1987; Horner, de Ricqlès & Padian, 1999; Chinsamy-Turan, 2005; Huttenlocker, Woodward & Hall, 2013). However, based on the increasing organization of more external tissues and a decrease in vascular density and anastomoses in these regions, both individuals had slowed linear growth from more rapid rates earlier in ontogeny, reaching the stage of depressed growth rates prior to asymptotic size within a few years, consistent with other studies of *D. romeri* histology (Werning, 2013). The size, histology, and ontogenetic ages of these smaller individuals is similar to other individuals of *D. romeri* that have been histologically sampled (Werning, 2013), suggesting that the relationship between size and histological signal is consistent across this taxon. The histology of the largest femur (GR 1038) suggests that this individual had nearly reached full-size. The subperiosteal portion of the cortex is composed of poorly vascularized parallel-fibered or lamellar bone with two to three closely spaced LAGs. If this is an EFS this may indicate that asymptotic size had been reached in this individual. However, the uneven spacing of the LAGs may suggest that this is not an EFS, but that the individual deposited a triple LAG shortly before death. Regardless, it is clear that linear growth had greatly slowed, if not completely ceased in this individual. Using these data in conjunction with the bone scar data (see below) we hypothesize that the size of the largest femur (GR 1038) provides a rough estimate on the maximum femoral size reached by *D. romeri* at skeletal maturity, although the individual may have grown slightly larger before reaching maximum size (especially if the three LAGs are not the onset of an EFS). The smaller femur also possesses some histological indicators of slowed growth (e.g., decreased vascularization), which could be indicative of intraspecific variation in maximum body size in this taxon, or that growth had only slowed in this individual as part of an annual cycle that ended with the deposition of a double LAG (Fig. 3A).

Given that the *M. iliofemoralis externus*, the insertion of which is hypothesized to form the trochanteric shelf of dinosauriforms (Hutchinson, 2001), does not form a distinct structure in *D. romeri*, the ‘trochanteric shelf’ of *D. gregorii* may have independently evolved in *D. gregorii* and dinosauriforms. Conversely, the trochanteric shelf may have been present in all skeletally mature lagerpetids except *D. romeri*, but the immaturity of all other known lagerpetid individuals prevents its observation. The muscles forming the anterior trochanter and trochanteric shelf (*Mm. iliotrochantericus caudalis* and *iliofemoralis externus*, respectively) have been hypothesized to be homologous with the crocodylian *M. iliofemoralis*, splitting from a single muscle into two at Dinosauriformes (Hutchinson, 2001). If the *M. iliofemoralis* insertion did shift proximally on the femur at the common ancestor of Dinosauromorpha, and only split into two heads in Dinosauriformes (Nesbitt et al., 2009a), then this may be supported by the simultaneous appearance of the anterior trochanter and ‘trochanteric shelf’ in the ontogeny of *D. gregorii*, as well as the continuous structure formed by this scar(s). However, the non-ornithodiran avemetatarsalian *Teleocrater rhadinus* and other aphanosaurs possess a proximally located attachment for *M. iliotrochantericus caudalis* and a more distally located attachment for *M. iliofemoralis externus* (Nesbitt et al., 2017). Therefore, the split of the ancestral *M. iliofemoralis* has already occurred in this taxon, with only one portion of the split shifting proximally. This could indicate that 1) the split of *M. iliofemoralis* occurred at Aphanosauria + Dinosauria, and by Dinosauromorpha, the *M. iliofemoralis externus* had also shifted proximally to insert next to *M. iliotrochantericus caudalis* in lagerpetids, with the continuous structure in *Dromomeron* representing the insertion of two muscles, or that 2) the split and proximal movement of *M. iliofemoralis* evolved independently in *Teleocrater* and Dinosauriformes. In the latter hypothesis, the anterior trochanter-trochanteric shelf structure of *D. gregorii* (and corresponding linear scars of other lagerpetids) would be the insertion of a single muscle, *M. iliofemoralis*. Both scenarios are plausible based on the phylogenetic relationships and optimizations of character states.

Based on these data, we hypothesize that the absence of an anterior trochanter and other scars of the proximal end of the femur in *D. romeri* is an autapomorphic feature of this taxon, and not simply indicative of the morphological immaturity of known individuals of *D. romeri*, falsifying the hypothesis (Bennett, 2015) that *D. romeri* is an immature *Tawa hallae*. The absence of a fourth trochanter (i.e., ridge for attachment of *M. caudifemoralis*) is especially striking. This feature is widely present among early archosaurs and is one of the first well-developed muscle scars to form during ontogeny, present before all other femoral scars in early avemetatarsalians (e.g., *Teleocrater*, Nesbitt et al., 2017; *D. gregorii*, Nesbitt et al., 2009a; *Ixalerpeton*, Cabreira et al., 2016; *Silesaurus*, Piechowski, Tałanda & Dzik, 2014; *Asilisaurus*, Griffin & Nesbitt, 2016a) and pseudosuchians (e.g., hatchling *Alligator*, Brochu, 1992). Furthermore, the anterior trochanter consistently appears early in post-natal ontogeny among early bird-line archosaurs (Nesbitt et al., 2009a; Griffin & Nesbitt, 2016a; Griffin, 2018), so even though the sampled individuals of *D. romeri* had not completely ceased growth, the absence of this feature in individuals that are close to reaching histological maturity suggests that it is absent in all individuals of this taxon, regardless of maturity.

### Comparison with other dinosauromorphs

Although many immature individuals of early bird-line archosaurs lack an anterior trochanter (e.g., *Dromomeron gregorii*, Nesbitt et al., 2009a; *Asilisaurus*, Griffin & Nesbitt, 2016a), the absence of an anterior trochanter and fourth trochanter throughout ontogeny is unique to *D. romeri* among known Triassic dinosauromorphs. *Lagerpeton chanarensis* possesses both a large, aliform fourth trochanter and an anterior trochanter in the form of a low rugosity (Sereno & Arcucci, 1994). Larger individuals of *D. gregorii* (Nesbitt et al., 2009a) possess a fourth trochanter and a ridge that is probably homologous with the ‘obturator ridge’ of early theropods and *Asilisaurus* (Griffin, 2018), as well as an anterior trochanter. *D. gigas* possesses a large anterior trochanter and ‘obturator ridge’, as well as a large scar marking the insertion of the *M. caudofemoralis longus*; only the proximal end of this scar is preserved, but it was interpreted as lacking a true fourth trochanter (Martínez et al., 2016), possessing a flat scar more similar to what we have described in *D. romeri*. Although *Ixalerpeton polesinensis* lacks an anterior trochanter, it possesses a robust aliform fourth trochanter, and a small scar similar in location (Cabreira et al., 2016: Fig. S1; ULBRA-PVT059) to the insertion of *M. caudofemoralis brevis* in dinosauriforms (Griffin & Nesbitt, 2016a; Griffin, 2018). Unfortunately, growth series are only known from *D. romeri* and *D. gregorii*, so broader comparisons between ontogenetic changes in femoral histology and gross morphology among lagerpetids are unavailable.

Intuitively, the morphology, size, and surface area of a muscle attachment would seem to be related to activity levels of the muscle, with larger or more robust scars indicating increased stress. However, this assumption has little empirical support: studies indirectly testing this assumption are often inconclusive and focused on humans (Foster, Buckley & Tayles, 2014), and the only experimental test of this hypothesis, in adult sheep, found no relationship between muscle activity and scar size (Zumwalt, 2006). Muscle activity is known to play a major role in the formation of overall bone morphology early in development, with paralyzed chick embryos possessing splayed and distorted limb bones (e.g., Hall & Herring, 1990; Hosseini & Hogg, 1991; Lamb et al., 2003), but this is a distinct process from the post-natal formation of well-developed bone scars, and the two may not be related. How muscle activity relates to the formation of muscle scars in non-mammalian taxa, or during ontogeny, remains poorly understood. Therefore, the relationship between these factors and archosaurian bone scars is unclear, so this lack of scarring in *D. romeri* is not evidence of a difference in activity level or body mass between this taxon and other lagerpetids. Whether differences in muscle scars relate to disparity in locomotion remains to be tested.

To date, there have been no histological investigations of any lagerpetids besides *D. romeri* (Werning, 2013; this study) because of a scarcity of material. However, the bone histology of the aphanosaur *Teleocrater* (Nesbitt et al., 2017), the early pterosaurs *Eudimorphodon*, *Dimorphodon*, and *Dorygnathus* (Padian, Horner & de Ricqlès, 2004), the silesaurids *Asilisaurus* (Griffin & Nesbitt, 2016a) and *Silesaurus* (Fostowicz-Frelik & Sulej, 2010), the possible dinosaur or close dinosaurian relative *Nyasasaurus* (Nesbitt et al., 2013), and many early dinosaurs (e.g., *Herrerasaurus ischigualastensis*, de Ricqlès, Padian & Horner, 2003; Padian, Horner & de Ricqlès, 2004; *Tawa hallae*, Werning, 2013; *Coelophysis bauri*, Colbert, 1995; Nesbitt et al., 2006; *Megapnosaurus rhodesiensis*, Raath, 1977; Chinsamy, 1990, Werning, 2013; *Lesothosaurus*, Knoll, Padian & de Ricqlès, 2010; *Scutellosaurus*, Padian, Horner & de Ricqlès, 2004) have all been described, allowing for broad comparison of the growth of *D. romeri* with other early avemetatarsalians. The histology of *Teleocrater* (fibula and humerus) and *D. romeri* (femora, tibia, and fibula) are broadly similar: both possess cortices consisting of woven bone with longitudinal vascular canals and primary osteons and are more similar to each other than to stem archosaurs (Botha-Brink & Smith, 2011; Werning, 2013). However, much of the outer cortex of *D. romeri* possesses more organized collagen fibers, osteocyte lacunae preferentially oriented circumferentially, lower vascularization, fewer anastomoses, and are more similar to the histology of stem archosaurs (e.g., Botha-Brink & Smith, 2011). In contrast, the cortex of sampled elements of *Teleocrater* possesses more disorganized woven-fibered bone with a higher number of anastomoses, especially in the humerus (Nesbitt et al., 2017). Additionally, unlike both *Teleocrater* and other descriptions of *D. romeri* histology (Werning, 2013), the fibula of the largest individual of *D. romeri* possesses secondary osteons, although this could be related to the element sampled (fibulae of *Dromomeron* have not been previously sampled), or the ontogenetic age of the individual. The histology of *Teleocrater* suggests a more sustained high rate of growth over a longer time. Whereas much of the outer cortex of *D. romeri* possesses features that suggest slowing growth (e.g., more organized collagen fibers, osteocyte lacunae preferentially oriented circumferentially, lower vascularization, fewer anastomoses), the cortex of sampled elements of *Teleocrater* possesses more disorganized and vascularized woven-fibered bone (Nesbitt et al., 2017). All these features suggest that, although at their highest rates of growth *D. romeri* and *Teleocrater* present similar histology, this rate of growth appears to be more sustained in *Teleocrater*. This shorter period of sustained growth in *D. romeri* may be related to its smaller body size—that is, *D. romeri* simply did not have to grow for as long to reach maximum size. This shorter and lower rate of sustained growth is also true in comparison with the histology of silesaurids, which possess larger regions of disorganized woven bone and more anastomosing vascular canals than are present in *D. romeri*, although the vascularization level of silesaurids is similar to that of the inner cortex of *D. romeri* (Fostowicz-Frelik & Sulej, 2010; Griffin & Nesbitt, 2016a).

The Late Triassic pterosaur *Eudimorphodon* possesses more organized bone tissues than *D. romeri*, with highly organized parallel-fibered tissue and a moderate level of vascularization with rarely anastomosing longitudinal canals (Padian, Horner & de Ricqlès, 2004; Werning, 2013), all indicating a slower rate of growth. However, because of the thin pterosaurian bone cortex, the presumably faster-growing tissue from earlier in ontogeny has been eroded, and confidently assessing the maturity of the sampled individual is difficult (Werning, 2013). *Dimorphodon*, a pterosaur from the Early Jurassic, possesses mainly disorganized woven-fibered bone similar to *Dromomeron romeri*, with some areas of more parallel-fibered bone. The degree of vascularization is slightly lower than that of *D. romeri*, but with a larger number of anastomosing vascular canals and looser tissue organization (de Ricqlès et al., 2000; Padian, Horner & de Ricqlès, 2004; Werning, 2013). These bone tissues suggest a higher rate of growth than that of *D. romeri.* The bone histology of the dinosauriform *Nyasasaurus* shows much higher levels of tissue disorganization, level of vascularity, anastomoses, and osteocyte density (Nesbitt et al., 2013) which is similar to that of early dinosaurs (e.g., *Herrerasaurus ischigualastensis*, de Ricqlès, Padian & Horner, 2003; Padian, Horner & de Ricqlès, 2004; *Tawa hallae*, Werning, 2013; *Coelophysis bauri*, Colbert, 1995; Nesbitt et al., 2006; *Megapnosaurus rhodesiensis*, Raath, 1977; Chinsamy, 1990, Werning, 2013; *Lesothosaurus*, Knoll, Padian & de Ricqlès, 2010; *Scutellosaurus*, Padian, Horner & de Ricqlès, 2004; see Werning, 2013 for a comparative review of early dinosaur osteohistology). Therefore, the bone histology of sampled early dinosaurs and their close relatives also suggests sustained growth proceeding more rapidly than in *D. romeri.*

All taxa discussed, including *D. romeri* and excepting *Eudimorphodon*, shift from predominantly disorganized woven-fibered to highly organized parallel-fibered bone tissue later in ontogeny as growth slows, and all but *Asilisaurus*, *Nyasasaurus*, and the pterosaurs deposit LAGs. Based on this, *Dromomeron romeri* appears to have possessed a growth rate most similar to *Teleocrater rhadinus*, although sustained over a shorter period of time, with a maximum rate of growth that was slower than most pterosaurs, dinosauriforms, and dinosaurs but faster than what is known for *Eudimorphodon*.

### Implications for assessing skeletal maturity in early avemetatarsalians

Although *Dromomeron romeri* lacks many femoral bone scars that are present in skeletally mature individuals of many other early avemetatarsalians, one scar—the attachment of *M. caudofemoralis longus*—does appear during post-natal ontogeny in this taxon. This is consistent with the general trend of increasing number and size of muscle scars with increasing skeletal maturity observed in other early avemetatarsalians (Nesbitt et al., 2009a; Piechowski, Tałanda & Dzik, 2014; Griffin & Nesbitt, 2016a, 2016b; Griffin, 2018) and in their closest living relatives (Brochu, 1992, 1996; Tumarkin-Deratzian, Vann & Dodson, 2006; 2007; Griffin & Nesbitt, 2016b). Therefore, the sampled series of *D. romeri* provides more evidence that the presence of bone scars is a reliable indicator of increasing skeletal maturity among archosaurs in general, and especially for early avemetatarsalians.

That said, the presence of a single bone scar or even a small set of scars may not be indicative that full skeletal maturity (i.e., cessation of linear growth; presence of all morphological features of maturity) has been reached in a given individual. Because bone scars appear in a stepwise pattern during ontogeny, and not simultaneously (Brochu, 1992; Tumarkin-Deratzian, Vann & Dodson, 2006, 2007; Nesbitt et al., 2009a; Piechowski, Tałanda & Dzik, 2014; Griffin & Nesbitt, 2016a, 2016b; Griffin, 2018), and because an individual may develop bone scars before histological maturity and attainment of asymptotic size (Griffin & Nesbitt, 2016a; this study), the presence of a single scar is a better indicator of relative skeletal maturity in comparison to conspecific individuals or those of close relatives, rather than as an absolute method of determining skeletal maturity. For example, an individual of *D. gregorii* possessing an anterior trochanter can be reasonably assumed to have progressed further in ontogeny than an individual lacking this scar. Because both taxa are closely related, and both are known to develop the anterior trochanter, an individual of *D. gigas* possessing this same scar can likewise be assumed to have attained a higher level of maturity than an individual of *D. gregorii* that lacks this scar. However, this does not indicate that all individuals of *D. gregorii* and *D. gigas* that possess the anterior trochanter are at the same ontogenetic stage or level of skeletal maturity.

**Figure 4:**
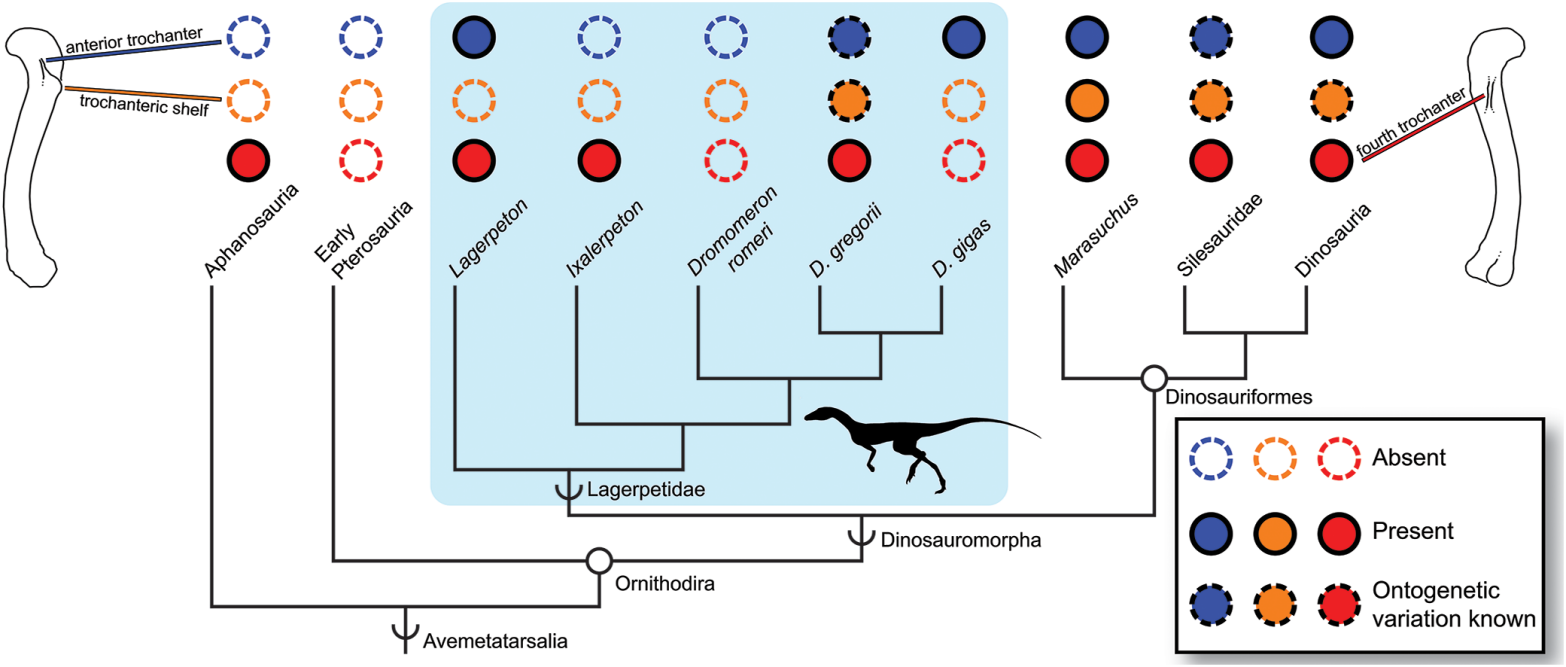
Distribution of the fourth trochanter, anterior trochanter, and trochanteric shelf among early bird-line archosaurs. Note that absences in Lagerpetidae may indicate either true absences (e.g., *Dromomeron romeri*) or immaturity of known individuals. Taxa for which these features are known to be ontogenetically variable are indicated. Silhouette from Cabreira et al. (2016: Fig. 1A). Femoral line drawings based on the *Dromomeron gregorii* holotype (TMM-31100-1306) by Christopher Griffin. Phylogeny follows Nesbitt et al. (2017) and Müller, Langer & Dias-da-Silva (2018).

Characters useful for maturity assessment vary across clades, and our confidence in assessment based on characters should vary with phylogenetic proximity to well-taxa with established growth patterns (e.g., Irmis, 2007). The anterior trochanter appears early in the ontogeny of dinosauriforms (Griffin, 2018), but in lagerpetids appears later in ontogeny (*D. gregorii*, Nesbitt et al., 2009a) or potentially not at all (*Ixalerpeton*, Cabreira et al., 2016, although this individual could be immature; *D. romeri*, this study), and is apparently absent in aphanosaurs (with a low mound marking this muscle insertion instead, Nesbitt et al., 2017). Therefore, the absence of an anterior trochanter in an individual may: 1) be uninformative for assessing skeletal maturity (in aphanosaurs); 2) indicate that the individual is skeletally immature, but with histological corroboration necessary (in lagerpetids); or 3) be strongly indicative that the individual is in the earliest stages of skeletal maturity (in dinosauriforms). This high degree of inter- and intraspecific variation in the anterior trochanter, trochanteric shelf, and potentially the fourth trochanter among early avemetatarsalians may be suggestive of a ‘zone of developmental variability’ (a complex pattern of character gains and losses before character fixation; see Bever, Gauthier & Wagner, 2011; Fig. 4). However, more work must be done to determine whether the absence of these scars in some lagerpetids is simply from the immaturity of individuals, or if there is true variability in the presence of these characters. Therefore, although the presence of muscle scars is indicative of increasing skeletal maturity, the phylogenetic history of each feature must be considered when assessing relative maturity (Fig. 4), and in some clades corroboration with other characters or osteohistology may be necessary.

Intraspecific variation in ontogenetic patterns may also complicate assessing maturity via gross morphology. There appear to be multiple sequences by which morphological characters progress from immature to mature states among silesaurids and early dinosaurs (Griffin & Nesbitt, 2016a, 2016b; Barta, Nesbitt & Norell, 2017; Griffin, 2018), so that the presence of a muscle scar or scars may not indicate the same level of maturity in all individuals, because the relative timing and body size at scar appearance may be widely disparate. However, whether this high intraspecific variation is constrained to Dinosauriformes or is more widespread among early diverging avemetatarsalians has not been explored. We did not observe similar levels of intraspecific variation among the growth series of *D. romeri*, either in gross morphology or histology as they relate to femoral size, and such variation has also not been reported in *D. gregorii*, the only other lagerpetid known from a growth series. Additionally, the five femoral specimens of *Lagerpeton chanarensis* are of roughly equivalent sizes, but also show no variation like that present in similarly sized early dinosauriform individuals (Nesbitt et al., 2009a). Therefore, we tentatively hypothesize that this high intraspecific variation in growth patterns is restricted to Dinosauriformes, although larger sample sizes of lagerpetid growth series will provide better testing of this hypothesis. Alternatively, if the intraspecific variation reported in some later pterosaurs (e.g., *Rhamphorhynchus*, Prondvai et al., 2012) is homologous with that in early dinosauriforms, this would imply that this high variation extends to at least the common ancestor of pterosaurs, lagerpetids, and dinosaurs.

## Conclusions

Unlike many other early avemetatarsalians, even the largest known individuals of *Dromomeron romeri* do not possess large, robust femoral bone scars. Because bone histology—generally consisting of more organized woven-fibered and parallel-fibered primary bone, longitudinal vascular canals, and one to two preserved LAGs—indicates that these large individuals are close to reaching asymptotic size, and are therefore near skeletal maturity, this absence of bone scars is not the result of the immaturity of the individuals in question. Although these features are important for assessing maturity in many groups, phylogenetic history must be considered when utilizing femoral scars to assess skeletal maturity. Complicating the matter is intraspecific variation—although there is currently no evidence in lagerpetids for levels of intraspecific variation as high as has been observed in dinosauriforms, small sample sizes may be hindering observation of this signal.

## Institutional abbreviations

GR: Ghost Ranch Ruth Hall Museum of Paleontology, Abiquiu, NM, USA.
TMM: Vertebrate Paleontology Laboratory, Texas Natural Science Center, Austin, TX, USA.
ULBRA: Universidade Luterana do Brasil, Coleção de Paleovertebrados, Canoas, Rio Grande do Sul, Brazil.

## Additional Information and Declarations

### Field Study Permissions

The following information was supplied relating to field study approvals (i.e., approving body and any reference numbers): Fieldwork at Ghost Ranch was conducted under permission from Ghost Ranch Conference Center.

### Data Availability

The following information was supplied regarding data availability: Morphobank (Project Number 3127: http://morphobank.org/permalink/?P3127).
